# Prognostic value of heart valve calcifications for cardiovascular events in a lung cancer screening population

**DOI:** 10.1007/s10554-015-0664-4

**Published:** 2015-05-12

**Authors:** Martin J. Willemink, Richard A. P. Takx, Ivana Išgum, Harry J. de Koning, Matthijs Oudkerk, Willem P. Th. M. Mali, Ricardo P. J. Budde, Tim Leiner, Rozemarijn Vliegenthart, Pim A. de Jong

**Affiliations:** Department of Radiology, University Medical Center Utrecht, E01.132, P.O. Box 85500, 3508 GA Utrecht, The Netherlands; Image Sciences Institute, University Medical Center Utrecht, Utrecht, The Netherlands; Department of Public Health, Erasmus Medical Center Rotterdam, Rotterdam, The Netherlands; Center for Medical Imaging - North East Netherlands, University Medical Center Groningen, University of Groningen, Groningen, The Netherlands; Department of Radiology, Erasmus Medical Center Rotterdam, Rotterdam, The Netherlands; Department of Radiology, University Medical Center Groningen, University of Groningen, Groningen, The Netherlands

**Keywords:** Lung cancer screening, Heart valves, Calcifications, Computed tomography, Prognosis, Cardiovascular events

## Abstract

To assess the prognostic value of aortic valve and mitral valve/annulus calcifications for cardiovascular events in heavily smoking men without a history of cardiovascular disease. Heavily smoking men without a cardiovascular disease history who underwent non-contrast-enhanced low-radiation-dose chest CT for lung cancer screening were included. Non-imaging predictors (age, smoking status and pack-years) were collected and imaging-predictors (calcium volume of the coronary arteries, aorta, aortic valve and mitral valve/annulus) were obtained. The outcome was the occurrence of cardiovascular events. Multivariable Cox proportional-hazards regression was used to calculate hazard-ratios (HRs) with 95 % confidence interval (CI). Subsequently, concordance-statistics were calculated. In total 3111 individuals were included, of whom 186 (6.0 %) developed a cardiovascular event during a follow-up of 2.9 (Q1–Q3, 2.7–3.3) years. If aortic (n = 657) or mitral (n = 85) annulus/valve calcifications were present, cardiovascular event incidence increased to 9.0 % (n = 59) or 12.9 % (n = 11), respectively. HRs of aortic and mitral valve/annulus calcium volume for cardiovascular events were 1.46 (95 % CI, 1.09–1.84) and 2.74 (95 % CI, 0.92–4.56) per 500 mm^3^. The c-statistic of a basic model including age, pack-years, current smoking status, coronary and aorta calcium volume was 0.68 (95 % CI, 0.63–0.72), which did not change after adding heart valve calcium volume. Aortic valve calcifications are predictors of future cardiovascular events. However, there was no added prognostic value beyond age, number of pack-years, current smoking status, coronary and aorta calcium volume for short term cardiovascular events.

## Introduction

Cardiovascular disease is one of the most important causes of death in heavy cigarette smokers [1]. Calcifications of the coronary arteries, aorta and heart valves are known predictors for cardiovascular events and can be quantified with electrocardiography (ECG) synchronized computed tomography (CT) [2–9].

Lung cancer screening studies such as the Dutch Belgian randomized lung cancer screening trial (NELSON) and the national lung screening trial (NLST) involve CT imaging of the chest without ECG synchronization [1, 10, 11]. These screening studies are aimed at detecting lung cancer, but they can also provide information about other diseases such as chronic obstructive pulmonary disease and cardiovascular disease [12, 13]. Despite the absence of ECG triggering, recent studies have shown that coronary and aortic calcifications can be quantified on lung cancer screening chest CT images and can provide prognostic information on future cardiovascular events [14–17]. Since smoking is not only a risk factor for lung cancer, but also for cardiovascular disease, these results could be beneficial for the effectiveness of chest CT screening in substantial smokers.

A previous study has shown that it is technically possible to detect and quantify cardiac valve calcifications on low-dose unenhanced non-triggered lung cancer screening CT images [18]. However, the prognostic value of heart valve calcifications for cardiovascular disease in heavily smoking men is unknown.

The aim of the current study was to assess the prognostic value of calcifications of the aortic valve and mitral valve/annulus for cardiovascular events in 3111 heavily smoking men without a history of cardiovascular disease, participating in the NELSON lung cancer screening trial.

## Materials and methods

### Study population

Heavily smoking or formerly smoking subjects without a history of cardiovascular disease who underwent a chest CT-scan as part of the NELSON screening trial (Clinical Trial Registration ISRCTN63545820) at the University Medical Centers of Groningen and Utrecht were included.

The NELSON trial was approved by the Dutch Minister of Health and the local Ethical Reseach Boards of the participating hospitals. The study population was recruited between September 2003 and April 2006. Questionnaires were sent to individuals living in four regions (Groningen, Utrecht, Haarlem, and Leuven) [10, 11]. The addresses of individuals aged between 50 and 75 were obtained from the population registries. Based on the questionnaires, 15,822 individuals with high lung cancer risk were included. Only individuals were included who had smoked 15 cigarettes or more per day during 25 years, or 10 cigarettes or more per day during 30 years, and were still smoking or had quit <10 years ago [10]. The study population was divided into a screening group of 7915 individuals who underwent four chest CT scans and a control group of 7907 individuals. Exclusion criteria included not enough cardiopulmonary reserve to undergo surgery, current or past renal cancer, melanoma or breast cancer, and diagnosis of lung cancer within 5 years before filling in the questionnaire and lung cancer that is still treated, and a chest CT scan within 1 year before filling out the questionnaire [11].

For the current study, subjects from the screening groups of two centers (Groningen and Utrecht) were included for efficiency reasons. Since only 89 women were present in this sample, women were excluded. All subjects underwent a baseline chest CT scan between January 2004 and December 2007.

### Image acquisition

Non-contrast enhanced images of the chest were acquired without ECG gating on 16-slice CT systems (Brilliance 16P or Mx8000 IDT, Philips Healthcare, Best, The Netherlands or Sensation 16, Siemens Healthcare, Forchheim, Germany). Low radiation dose protocols were used with a collimation of 16 × 0.75 mm, a tube voltage of 120 kV for participants weighing less than 80 kg or 140 kV for participants weighing 80 kg or more. Tube current settings varied based on hardware and participant weight.

### Data collection

Information about non-imaging predictors (age, smoking status and pack years) was collected from the baseline NELSON questionnaires. Calcium volume of the aorta and coronary arteries were previously obtained using an in-house developed software package as described by Isgum et al. [19, 20] and Mets et al. [15]. This software package was also used in the current study for quantification of aortic and mitral valve calcifications. Calcium volume of the aortic valve and the mitral valve/annulus were quantified manually. Since it is very difficult to differentiate the mitral annulus from the mitral valve on a non-contrast enhanced low radiation dose non-gated chest CT, the values were taken together as total mitral valve calcification. In the remainder, ‘mitral valve calcification’ refers to calcification of both valve and annulus (if applicable). The volume (mm^3^) of regions with a density of 130 Hounsfield units or more was determined. If no calcifications were present, the volume was set at 0 mm^3^. A previous study by van Hamersvelt et al. [18] has shown that the inter-observer and inter-examination variability of cardiac valve measurements is sufficient to allow for longitudinal studies, especially for the aortic valve.

The outcome variable was the occurrence of cardiovascular events, which was obtained by linkage of subjects with the National Death Registry and the National Registry of Hospital Discharge Diagnoses. Both fatal and nonfatal cardiovascular events that occurred between January 1995 and January 2008 were included. Cardiovascular events were classified according to the World Health Organization’s International Classification of Diseases [21]. Cardiovascular events included hypertensive disease, ischemic heart disease, heart failure, diseases of arteries, arterioles and capillaries, cerebrovascular disease, or other heart disease. Individuals with a history of cardiovascular disease, defined as a hospitalization for a cardiovascular event in the 10 years before the CT, were excluded. If a subject was admitted to a hospital for a cardiovascular disease and died later on due to a cardiovascular disease, cardiovascular death was chosen as the outcome value. If a subject was admitted multiple times to a hospital for a cardiovascular disease, the diagnosis at first discharge was used.

### Data analysis

Missing data were imputed using single linear regression imputation since less than 5 % of data were missing and missing data were random. Baseline characteristics were compared between individuals with a cardiovascular event and individuals without a cardiovascular event using the Mann–Whitney U test (continuous data) or the Chi square test (frequency data). Calcification volumes of the aorta and coronaries and the aortic and mitral valves were truncated at the 99th percentile as previously described [15]. Furthermore, the number of pack years was truncated at 50 years since higher numbers of pack years did not increase cardiovascular risk [15]. In order to perform a Cox proportional hazards (PH) regression analysis, the PH assumption was checked by the Schoenfield residuals. A multivariable prediction model was made for cardiovascular risk using Cox PH regression analysis. Based on this prediction model the hazard ratios (HRs) of the predictors were calculated with 95 % confidence intervals (CI). Subsequently, a bootstrap analysis was performed to correct for over-optimism. An age-adjusted survival curve was evaluated by presence and absence of aortic valve calcifications.

Concordance-statistics (c-statistics) were calculated to evaluate the discriminative capacity between a subject with and a subject without a cardiovascular event [22, 23].

Data are presented as medians (first quartile–third quartile) unless otherwise stated. Statistical analyses were performed using R version 3.0.2 (R Foundation for Statistical Computing, Vienna, Austria) and SPSS Statistics version 20 (SPSS Inc, Chicago, Illinois, The United States). Statistical testing was two-sided, and a *p* value below 0.05 was considered statistically significant.

## Results

**Fig. 1:**
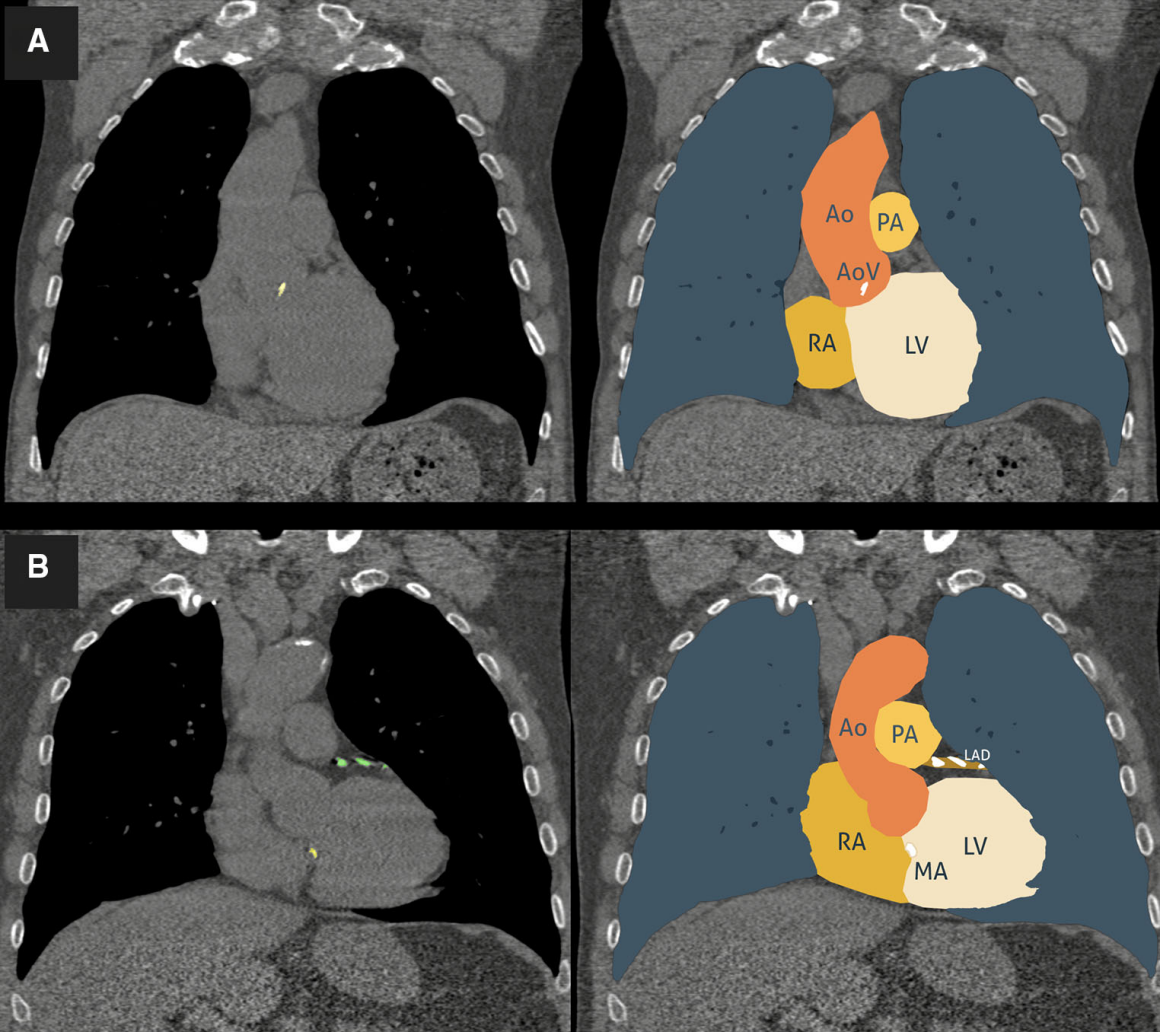
Subject with a calcified aortic valve (**a)**, and a subject with coronary and mitral annulus calcifications (**b**) *Ao* aorta, *AoV* aortic valve, *PA* pulmonary artery, *RA* right atrium, *LV* left ventricle, *MA* mitral annulus, *LAD* left anterior descending coronary artery

In total 3111 men were included, 1620 from the region of Groningen and 1491 from the region of Utrecht. Detailed characteristics of the individuals are listed in Table 1. In total 0.13 % of data for 6 variables was missing. Therefore, missing data were imputed using single linear regression imputation. In total 186 individuals (6.0 %) developed a cardiovascular event during a follow-up of 2.9 (Q1–Q3, 2.7–3.3) years. Aortic valve calcifications were present in 657 individuals (21.1 %) of whom 59 individuals (9.0 %) developed a cardiovascular event. An example image of a subject with a calcification in the aortic valve is displayed in Fig. 1. Mitral valve calcifications were present in 85 individuals (2.7 %) of whom 11 developed a cardiovascular event (12.9 %).

**Table 1:** Demographic characteristics of the 3111 lung cancer screening participants

Variable	No event (N = 2925)	CV event (N = 186)	*P*-value
Age (years)	59.2 (55.9–63.3)	60.8 (57.1–65.1)	0.000*
Length height (cm)	179.0 (174.8–183.0)	178.0 (175.0–183.0)	0.560*
Number of current smokers (%)	1658 (56.7 %)	128 (68.8 %)	0.000**
Pack years	38.0 (28.0–46.2)	38.7 (29.7–49.5)	0.019*
Coronary calcification volume (mm^3^)	126.4 (6.8–528.6)	590.2 (152.1–1441.4)	0.000*
Aortic calcification volume (mm^3^)	419.4 (74.0–1365.9)	1352.7 (301.7–3128.6)	0.000*
Number of individuals with aortic valve calcifications	598 (20.4 %)	59 (31.7 %)	0.000**
Aortic valve calcification volume (mm^3^)	96.3 (39.7–235.4)	179.1 (71.6–387.1)	0.002*
Number of individuals with mitral valve/annulus calcifications	74 (2.5 %)	11 (5.9 %)	0.006**
Mitral valve/annulus calcification volume (mm^3^)	140.8 (54.4–357.1)	216.9 (44.5–356.4)	0.977*

Values are medians (first quartile–third quartile) or numbers (percentages)

Significant values are marked bold

*P-value based on Mann–Whitney U test

**P-value based on Chi square test

The plotted Schoenfeld residuals showed no relevant deviations and thus the PH assumption was not violated and therefore a multivariable Cox PH regression analysis could be performed. For each variable the HR was calculated with 95 % CI. Bootstrap analysis resulted in an over-optimism correction factor of 0.988. Corrected HRs with 95 % CI are listed in Table 2. Significant predictors included current smoking status [HR for not smoking of 0.82 (0.72–0.93)], coronary calcium volume per 500 mm^3^ [HR of 1.11 (95 % CI, 1.06–1.16)], aortic calcium volume per 500 mm^3^ [HR of 1.02 (95 % CI, 1.00–1.04)] and aortic valve calcium volume per 500 mm^3^ [1.46 (95 % CI, 1.09–1.84)]. Mitral valve calcification volume was not a significant predictor with an HR of 2.74 [95 % CI, 0.92–4.56] per 500 mm^3^.

**Table 2:** Effects of predictors on 3 year risk on cardiovascular events (N = 3111)

Predictor	Beta	SE	*P* value	Hazard ratio (95 % CI)
Age (per 10 years)	0.223	0.149	0.1295	1.25 (0.96–1.54)
Former smoker	−0.193	0.054	**0.0003**	**0.82 (0.72–0.93)**
Pack years (per 10 years)	0.118	0.075	0.1125	1.13 (0.98–1.27)
Coronary calcium volume (per 500 mm^3^)	0.102	0.027	**0.0001**	**1.11 (1.06–1.16)**
Aortic calcium volume (per 500 mm^3^)	0.021	0.010	**0.0423**	**1.02 (1.00–1.04)**
Aortic valve calcium volume (per 500 mm^3^)	0.381	0.191	**0.0430**	**1.46 (1.09–1.84)**
Mitral valve/annulus calcium volume (per 500 mm^3^)	1.007	0.928	0.2718	2.74 (0.92–4.56)

Corrected for over-optimism with a correction factor of 0.988

Significant values are marked bold

**Fig. 2:**
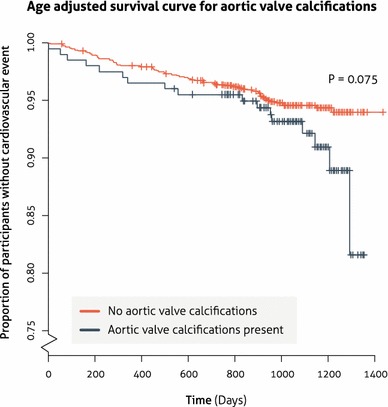
Survival curve for aortic valve calcifications adjusted for an age category of 55–60 years

An age-adjusted survival curve was evaluated by presence and absence of aortic valve calcifications (Fig. 2). The curve indicates that the presence of heart valve calcifications translates in more events. The c-statistic of a basic model with predictors proposed by Mets and colleagues [15] including age, pack years, current smoking status, coronary calcium volume and aorta calcium volume was 0.68 (95 % CI, 0.63–0.72). Adding both aortic valve and mitral valve calcium volume to this basic model resulted in a similar c-statistic of 0.68 (95 % CI, 0.64–0.73).

## Discussion

We evaluated the prognostic value of aortic valve and mitral valve/annulus calcifications for cardiovascular events in male lung cancer screening participants without a history of cardiovascular disease. The current study showed that calcifications of the aortic valve increased cardiovascular risk by almost 50 %. However, adding cardiac valve calcium volumes did not substantially improve a model proposed by Mets et al. [15] with age, number of pack years, current smoking status, coronary calcium volume and aorta calcium volume. Therefore, assessment of cardiac valve calcifications on lung cancer screening CT examinations is not recommended for 3 years cardiovascular risk assessment if calcifications of the coronary arteries and aorta are also evaluated.

Lung cancer screening with non-gated unenhanced chest CT is primarily aimed at lung cancer detection. However, these CT examinations can also provide information about other diseases including cardiovascular diseases, especially since smoking is not only a risk factor for lung cancer, but also for cardiovascular diseases [13, 15]. The current study showed that cardiac valve calcifications were significantly more prevalent in individuals who developed a cardiovascular event compared to individuals without an event (31.7 vs 20.4 % for aortic valve calcifications and 5.9 vs 2.5 % for mitral valve/annulus calcifications, respectively). These numbers are comparable to previously reported prevalence numbers of 18–27 % for aortic valve calcifications in routine clinical care patients and an elderly population based study [8, 24, 25] and 8–9 % for mitral valve calcifications in routine clinical care patients [24, 26]. If cardiac valve calcifications were present, the volumes of these calcifications were also significantly larger in individuals who developed a cardiovascular event (179.1 vs 96.3 mm^3^ for aortic valve calcifications and 216.9 vs 140.8 mm^3^ for mitral valve/annulus calcifications, respectively). This indicates a potential prognostic value of cardiac valve calcifications in a lung cancer screening setting, which was confirmed by our multivariable cox PH regression analysis. However, cardiac valve calcification volumes do not have added predictive value after 3 years follow-up beyond age, pack years, smoking status, coronary calcium volume and aorta calcium volume. This may be explained by the relatively short follow-up time of the current study. Probably calcifications of the coronaries and the aorta are associated with cardiovascular events on the short term, whereas heart valve calcifications may be associated with more chronic diseases. Another explanation may be the etiology of cardiac valve calcifications that shares common pathways and risk factors with coronary artery calcification. Some studies indicate that calcifications of the coronary arteries, the aorta and the cardiac valves are all part of the progression of coronary atherosclerosis [27–29]. It is suggested that especially micro calcifications in the coronaries and aorta are part of the atherosclerotic pathway [30]. These may lead to plaque rupture and subsequently myocardial infarction or stroke [30]. Besides part of the atherosclerosis pathway, cardiac valve calcifications may also be a cause of valve failure [31]. Large calcifications of the aortic valve for example, may lead to impaired leaflet function followed by heart failure [30]. Calcifications of the mitral annulus are part of a chronic non-inflammatory degenerative process [32], and severe calcifications of the mitral annulus may cause mitral regurgitation [31]. Therefore, cardiac valve calcifications should be reported in the clinical radiology routine where individual risk factors may be unknown [33, 34].

The current study confirms and expands the results of Gondrie et al. [24]. They also evaluated the prognostic value of cardiac valve calcifications on future cardiovascular events. However, they evaluated a more heterogeneous routine care population with routine radiation dose chest CT exams (both with and without contrast medium) and they used a subjective assessment instead of the quantitative volume measurements as used in the current study. Gondrie et al. also found increased HRs for cardiac valve calcifications, but similar to our results cardiac valve calcifications lost their prognostic value for cardiovascular events when modelled with calcifications of the coronaries and aorta. They also had a relatively short follow-up.

An in-house developed software package was used for quantification of calcifications. Cardiac regions with Hounsfield units of 130 and higher were detected as calcium with this software package. This is similar to the validated Agatston score. However, the Agatston score was developed and validated for quantifying coronary calcifications on dedicated cardiac CT and not for cardiac valve calcifications on non-gated low-dose chest CT. Therefore, calcium quantification may be affected due to motion artifacts and worse image quality. However, newer CT systems result potentially in less artifacts and better image quality as compared to the 16-slice CT systems that are used in the current study.

Our study has limitations that should be addressed. First, despite the large sample size of the current study, only men and only (previous) smokers were included. Results may thus not be generalizable to women and non-smoking individuals. The latter is less relevant since this study is focused on additional information provided by lung cancer screening CT exams. Second, since not all Framingham risk score determinants were provided by the NELSON trial, the added value of cardiac valve calcifications beyond the Framingham risk score could not be assessed. However, we did include coronary artery calcium in our analyses, a direct measure of vascular disease, which has been proven to be a strong predictor for future cardiovascular events [35, 36], and is considered a reflection of the impact of known and unknown risk factors on the arterial wall. Third, our follow-up time was relatively short and therefore the emphasis was more on acute than on chronic disease. It may be that heart valve calcifications lead to cardiovascular events in the long term. Fourth, the NELSON lung cancer screening trial was designed and powered for lung cancer screening detection and not for cardiovascular events. Fifth, not only hard events but also soft events were included as outcome measures. Finally, the confidence interval for the HR of mitral valve/annulus calcification volumes was wider compared to aortic valve calcification volumes. This is presumably caused by the low prevalence of mitral valve/annulus calcifications (2.7 %) compared to the higher prevalence of aortic valve calcifications (21.1 %). Therefore, only a trend towards increased risk on future cardiovascular events could be evaluated for mitral valve/annulus calcifications. Moreover, van Hamersvelt et al. [18] showed that the reproducibility of mitral valve/annulus calcifications on lung cancer screening chest CT exams is not as good as for aortic valve calcifications. This may have contributed to weaker results for mitral valve/annulus calcifications as compared to aortic valve calcifications.

In conclusion, calcifications of the aortic valve are predictors for future cardiovascular events. However, there was no added prognostic value of cardiac valve calcium volumes beyond age, number of pack years, current smoking status, coronary calcium volume and aorta calcium volume for 3 years cardiovascular outcome.

## Abbreviations

CT: Computed tomography
C-statistics: Concordance statistics
CI: Confidence interval
NELSON: Dutch Belgian randomized lung cancer screening trial
ECG: Electrocardiography
HRs: Hazard ratios
NLST: National lung screening trial
PH: Proportional hazards
